# Effects of saponins R_b1_ and R_e_ in *American ginseng* intervention on intestinal microbiota of aging model

**DOI:** 10.3389/fnut.2024.1435778

**Published:** 2024-09-13

**Authors:** Mao Shi, HongXiu Fan, HongCheng Liu, YanRong Zhang

**Affiliations:** ^1^College of Food Science and Engineering, Jilin Agricultural University, Changchun, China; ^2^Jilin Provincial Center for Disease Control and Prevention, Changchun, China

**Keywords:** aging, *American ginseng*, R_b1_, R_e_, intestinal microbiota

## Abstract

Aging brings about physiological dysfunction, disease, and eventual mortality. An increasing number of studies indicate that aging can easily lead to dysbiosis of the gut microbiota, which can further affect digestion, nerves, cognition, emotions, and more. Therefore, gut bacteria play an important role in regulating the physical functions of aging populations. While saponins, the primary components of *American ginseng*, are frequently utilized for treating common ailments in the elderly due to their potent antioxidant properties, there is a scarcity of comprehensive studies on aging organisms. This study focused on 18 month old aging mice and investigated the effects of single intervention and combined intervention of R_b1_ and R_e_, the main components of Panax quinquefolium saponins, on the gut microbiota of aging mice. High throughput 16s RNA gene sequencing analysis was performed on the gut contents of the tested mice, and the results showed that R_b1_ and R_e_ had a significant impact on the gut microbiota. R_b1_, R_e_, and R_b1_ + R_e_ can effectively enhance the diversity of gut microbiota, especially in the combined Rb1 + Re group, which can recover to the level of young mice. Re can promote the abundance of probiotics such as Lactobacillus, Lactobacillaceae, and Lactobacillus, and inhibit the abundance of harmful bacteria such as Enterobacteriaceae. This indicates that the intervention of R_b1_, R_e_, and R_b1_ + R_e_ can maintain the homeostasis of gut microbiota, and the combined application of R_b1_ + R_e_ has a better effect. The relationship between aging, brain gut axis, and gut microbiota is very close. Saponins can improve the gut microbiota of aging individuals by maintaining the balance of gut microbiota and the normal function of the brain gut axis, enabling the body to achieve a gut microbiota homeostasis closer to that of young healthy mice.

## 1 Introduction

*American ginseng* (*Panax quinquefolius*) is a perennial herb belonging to the *Ginseng genus* in the *Ginkgoaceae* family (1). Indigenous to the deciduous woodlands of eastern North America, it has been widely cultivated worldwide (2). Extensive research has demonstrated the efficacy of *Panax quinquefolius* in regulating blood pressure, boosting immunity, reducing blood glucose levels, mitigating inflammation, and safeguarding the cardiovascular system, owing to its antioxidant properties (3). Saponins, the pivotal constituents of *Panax quinquefolius*, play a crucial role in positive immunomodulation, oxidative regulation, blood glucose control, and anti-radiation effects (4). Among the key saponin components, R_b1_, R_e_, R_d_, R_g1_, and R_b3_ collectively constitute over 70% of the total saponin content (5). Some of these components have been revealed to inhibit cancer cell growth, act as antioxidants, lower blood glucose levels, activate estrogen receptors, and enhance memory, among other beneficial effects (6). Furthermore, the saponin composition of *Panax quinquefolius* closely resembles that of ginsenosides, exhibiting similar efficacy in various aspects. As a result, *Panax quinquefolius* is often compared to traditional ginseng (7). Aging is an unavoidable biological phenomenon characterized by deteriorative changes in an individual’s functionality as they age. Ultimately, these changes compromise the organism’s ability to sustain normal functioning, culminating in eventual mortality (8). Numerous studies have indicated that the principal component of ginseng has the potential to extend lifespan, as demonstrated in models like Drosophila and Cryptobacterium hidradii nematodes (9). Additionally, the *Panax quinquefolius* extract CVT-E002 has been shown to prolong the lifespan of infants and juvenile mice with leukemia (10). Ginsenosides, non-starch polysaccharides from ginseng, and certain phenolic compounds exhibit antioxidant effects, with even ginseng leaf essential oil demonstrating free radical scavenging activity (11). The antioxidant properties of ginseng extracts have captured the interest of researchers, revealing that these extracts can shield cells from AA+ iron-induced reactive oxygen species (ROS) production and mitochondrial damage by activating AMP-activated protein kinase (AMPK). At the molecular level, ginseng is capable of initiating LKB1-dependent AMPK activation, contributing to enhanced cell survival (12).

The gut microbiota, including a large number of various microorganisms residing in the gastrointestinal tract, plays an important role as a component of the host system in maintaining the stability of the gastrointestinal environment and regulating host metabolism. Research has shown that each individual has at least 160 taxonomic species, belonging to 1,000–1,150 popular bacteria; their collective genome (“microbiota”) contains at least 100 times the number of genes in the genome (13). The composition of microbial communities develops in the first few years of life and is relatively stable in adulthood (14). The gut microbiota may be influenced by host genetics, health, diet, aging, and probiotics (15, 16). The changes in gut microbiota composition related to aging may be related to many diseases and disorders in the elderly (17). Recent studies have also shown that gut microbiota, especially certain specific strains, may enhance cell-mediated immunity in host animals, thereby altering age-related immune aging (18). As is well known, aminobutyric acid (GABA) is the most important inhibitory factor in the central nervous system of humans and other mammals, participating in neurotransmitters and metabolic processes in the brain. Research has shown that the levels of GABA in the gut are closely related to those in the central nervous system, and bifidobacteria and lactobacilli in the gut are involved in the production of GABA. Therefore, bifidobacteria and lactobacilli in the gut are closely related to some brain dysfunction, such as depression, cognitive impairment, synaptic dysfunction, etc. (19). Compared to fecal samples from young subjects, the proportion of Clostridium, Streptococcus, and Staphylococcus in fecal samples from elderly individuals has increased (20), which are commonly believed to have adverse effects on the body.

In addition, changes in the gut microbiota associated with aging can damage the host’s immune homeostasis, leading to pro-inflammatory responses, the formation of a malignant inflammatory cycle, and the possible occurrence of age-related diseases (21). Slowing gastrointestinal peristalsis or constipation in elderly people can reduce the proportion of intestinal bacteria excreted from the body, leading to the accumulation of pathogenic microorganisms in the intestine and causing intestinal bacterial overload (22). Aging can also affect the motor and perceptual functions of the gastrointestinal tract (23). The increase in age will be accompanied by changes in gastrointestinal function, such as decreased esophageal peristaltic pressure leading to swallowing difficulties and reduced colon motility leading to constipation (24), which will further increase the burden on the intestines of elderly people. The study by Kim et al. (25) suggests that age-related intestinal homeostasis disorders in model organisms can trigger congenital immune responses and chronic low-grade inflammation, leading to degenerative diseases and unhealthy aging. The study of gut microbiota and the gut brain axis, as well as their neural related functions, has received widespread attention in recent years. Therefore, the homeostasis of gut microbiota is closely related to the recovery of cognitive function in aging mice. The imbalance of gut microbiota is a key trigger for many diseases, including digestive system diseases directly associated with it, cardiovascular and neurological diseases indirectly associated with it. Therefore, having a healthy gut not only enriches the variety of gut microbiota, but also has a positive effect on the elderly population.

According to the previous research results of our team, saponins have a positive effect on aging mice and can improve the adverse effects of aging in multiple aspects such as immunity and cognition. Meanwhile, previous studies have found that dysregulation of the gut microbiota is closely related to neurological disorders such as Alzheimer’s Disease (AD), Parkinson’s Disease (PD), Huntington’s Disease (HD), and multiple sclerosis (MS) (26, 27). We do not know whether saponins have a positive effect on the gut microbiota of aging mice. Therefore, this study used 16s RNA sequencing technology to analyze the intestinal contents of mice, in order to reveal the effects of saponin monomer R_b1_ and R_e_ intervention alone and in combination on the intestinal microbiota of mice. In short understanding the changes in gut microbiota in aging mouse models is of great significance for understanding the mechanism of saponin action.

## 2 Materials and methods

### 2.1 Materials and reagents

Pharmaceutical grade saponins R_b1_(CDCV-ASB-00007192-101,purity≥98%) and R_e_(CAS:52286–59-6,purity≥98%) were purchased from the Natural Medicine Chemistry Laboratory of Jilin University and stored at 4°C according to the drug instructions. Aging model mice (C57BL/6,18 months old) were purchased from Changchun Yishi Experimental Animal Technology Co., LTD., and raised in Jilin Provincial Health Inspection Center (approved by the Experimental Animal Management Committee of Jilin Provincial Center for Disease Control and Prevention (JCDC [2023] No. 1)). DNA extraction kit, Phusion Flash High Fidelity PCR Premix, DNA Library Construction Kit, Purchased from ThermoFisher.

### 2.2 Grouping and administration of experimental mice

Forty healthy male C57BL/6 mice (18 months old, weight 28-32 g) were selected. The aging mouse models were put into the mouse house for 7 days, then they were divided into 4 groups on average, with 10 mice in each group. They were divided into Aged model group, R_b1_ administration group (R_b1_), Re administration group (R_e_) and R_b1_, R_e_ administration group (R_b1_ + R_e_). The dosage was formulated according to the optimal concentration of the team’s previous trial, that is, the dosage of R_b1_ was 30 mg/kg, and the dosage of R_e_ was 15 mg/kg. In addition, 10 6-month-old male healthy C57BL/6 mice (weight 20-25 g) were used as a control group (Col).

The mice were raised in Jilin Provincial Health Inspection Center in a constant temperature (21 ± 2°C) and 50% humidity environment, and the light was set to 12 h dark light cycle, and they were free to drink and eat. The saponin monomer drug was dissolved in distilled water according to the experimental design, and the mice were given the drug through drinking water every day. Control and aging groups drank distilled water. Before feeding, each mouse was starved for 12 h and then numbered and weighed. After 8 weeks of continuous feeding, each mouse was starved for 12 h, and then weighed. The mice were killed 20 h after the last administration and the relevant indexes were detected (28).

### 2.3 Laboratory mouse anatomy

The mouse model was anesthetized with 0.5% pentobarbital sodium. After anesthesia, all mice were subjected to a cesarean section experiment, and the intestinal segments from the jejunum to the ileum were cut off. Then, a scraper was used to sample the contents of the duodenum. All samples were immediately frozen in a−80°C freezer after collection. The remaining biological tissues after dissection were treated harmlessly.

### 2.4 16s rRNA gene sequence amplification

This study used the V3-V4 region of the 16s rRNA gene for detection. The primers used for amplification were the universal 520F universal primer (5‘- AYTGGGYDTAAAGNG-3’) and the 802R universal primer (5‘- TACNVGGTATCTAATCC-3’). Amplification was performed using Taq high fidelity enzyme. The PCR amplification system is shown in the Table 1 as follows:

**Table 1:** PCR amplification system.

Reagent	Concentration	Volume (μL)
d NTP	2.5 mmol·L^−1^	2
Phusion Flash High Fidelity PCR Premix	5×	5
520F primer	10μmmol·L^−1^	1
802R primer	10μmmol·L^−1^	1
DNA template	0.2 ng·μL^−1^	4
dd Water	——	12

PCR amplification conditions: pre denaturation at 98°C for 30 s; Then deform at 98°C for 30 s, anneal at 50°C for 30 s, and extend at 72°C for 30 s, a total of 25 cycles; Finally, extend at 72°C for 5 min. The PCR product gel was recovered and subjected to 16s rDNA V3-V4 sequencing by Tianjin Nuohe Zhiyuan Biotechnology Co., Ltd.

### 2.5 Sequencing data analysis

#### 2.5.1 Data quality control

After data sequencing is completed, raw data is obtained, which includes sequences with lower sequencing quality, sequences with splices, and sequences with a higher proportion of N. Raw data must first go through data quality control to obtain high-quality data before proceeding with subsequent analysis. In this study, FastQC 0.11.9 (29) was used for data quality control under the following conditions: (1) Remove reads containing adapters; (2) Remove reads with a N content ratio greater than 10%; (3) Remove reads containing all A bases; (4) Remove low-quality reads (alkaline bases with a quality value Q ≤ 20 account for more than 50% of the entire reads). The data after quality control is clean data.

#### 2.5.2 Sequence assembly and OTU classification

Use Qiime 2 (30) software to assemble all clean reads, divide them into an Operational Taxonomic Unit (OTU) based on 97% similarity, and then select the longest sequence of each class as the representative sequence. Then, use Qiime 2 to perform Blast alignment of all OTU sequences with Nr and Nt databases to obtain sequence annotation information for each OTU. Next, remove all sequencing samples with OTU abundance ratios below 0.001% to obtain effective OTUs. Then all obtained OTUs are sorted in descending order of abundance.

#### 2.5.3 Alpha diversity index analysis

Alpha diversity refers to the diversity within a specific region or ecosystem, which is a comprehensive indicator reflecting richness and evenness. This study analyzed the Chao1 index, Shannon index, ACE index, and Simpson index in Alpha diversity. Chao1 is commonly used in ecology to estimate the total number of species; The ACE index can estimate the actual number of species present in a community, and the larger the ACE index, the higher the richness of the community; The Shannon index comprehensively evaluates the richness and evenness of communities. The higher the Shannon index value, the greater the diversity and complexity of information in the system (31). Comprehensively evaluate the Alpha diversity index in each group to evaluate the diversity and evenness of gut microbiota in each group.

#### 2.5.4 Beta diversity index analysis

Beta diversity analysis is a comparison of diversity between different ecosystems, which is the rate of change in species composition along environmental gradients or between communities, used to represent the response of biological species to environmental heterogeneity. In this study, the Beta diversity analysis used the PCoA index to evaluate the similarity or dissimilarity of the study sample community composition.

#### 2.5.5 Species composition analysis

Based on the annotation information, this study analyzed the community structure of each observed sample at different classification levels. When comparing the community structure analysis of multiple samples together, one can also observe their changes. Here, we use bar charts to visualize the species composition of different groups, and use bar charts to visualize the bacterial content of the same sample (or unified group). This study analyzes the species composition of each group from five levels: phylum, class, order, family, genus, etc.

#### 2.5.6 Analysis of differences in species composition

In this study, species composition differences were analyzed using LEfSe (32). LDA Effect Size analysis can achieve comparison between multiple groups, and internal subgroup comparison analysis can be carried out to identify species with significant differences in abundance between groups. According to its analysis principle, it can be divided into three steps. The analysis principle is as follows: (1) Firstly, the parameter test ANOVA test is used in multiple group samples to detect species with significant differences in abundance between different groups, and the threshold is set to 0.05; (2) Then, the significantly different species obtained in the previous step were subjected to intergroup difference analysis using Wilcoxon rank sum test, with a threshold set at 0.05; (3) Finally, linear discriminant analysis was used to perform dimensionality reduction analysis on the data, and the influence of species with significant differences (i.e., LDA score) was evaluated. In this study, the LDA threshold was set to 3.5.

### 2.6 Joint data analysis

Usually, we conduct cluster analysis by studying the community function of gut microbiota, enrich OTUs based on the function of microbial abundance, and display them in the form of heatmaps. Heat maps are drawn using TBTools software. In addition, this study conducted redundancy analysis (RDA) based on significantly correlated microbial environmental factors, revealing the relationship between environmental variables and community species composition through dimensionality reduction. Used to visualize and quantify the association between multiple environmental variables and multivariate response variables (such as species abundance or presence/absence). Then, using JSD distance and Partition Around Medoids (PAM) clustering algorithm for intestinal type analysis, the samples are clustered based on relative species abundance. Use the Calinski Harabasz index to evaluate the results and determine the optimal number of clusters.

### 2.7 Data processing and analysis

SPSS 19.0 was used for statistical analysis. The two sets of data were directly compared with the practical one-way ANOVA or T-test. Three or more sets of data were compared for analysis using ANOVA. Data are expressed as mean ± standard deviation of no less than 3 biological replicates. The significance level was significant with *p* < 0.05.

## 3 Results

### 3.1 Data quality control results

All sequencing data were subjected to data quality control, and the results showed that the data quality in this study was good. Offline data is obtained by removing barcode and primer and concatenating them to obtain raw tags. Raw tags are further removed from chimeras and short sequences to obtain high-quality clean tags. The results show that the proportion of clean data is higher than 96.11%, and the average proportion of data is 98.16%.

The specific results are shown in the Table 2.

**Table 2:** Statistics of assembly results.

SampleID	Raw_tags	Clean_tags	Percentage (%)
Aged-r1	77,550	75,391	97.21
Aged-r2	71,095	69,064	97.11
Aged-r3	75,842	73,497	96.91
Aged-r4	52,645	51,124	97.11
Col-r1	33,564	33,105	98.61
Col-r2	46,231	45,380	98.21
Col-r3	34,314	33,733	98.31
Col-r4	58,826	57,718	98.11
R_b1_-r1	61,093	59,966	98.21
R_b1_-r2	42,075	41,438	98.51
R_b1_-r3	56,930	56,088	98.51
R_b1_-r4	71,199	70,017	98.31
R_e_-r1	80,920	79,497	98.21
R_e_-r2	52,783	52,134	98.81
R_e_-r3	56,543	55,876	98.81
R_e_-r4	62,577	61,431	98.21
R_b1_ + R_e_-r1	31,441	30,983	98.51
R_b1_ + R_e_-r2	49,425	48,650	98.41
R_b1_ + R_e_-r3	47,315	46,641	98.61
R_b1_ + R_e_-r4	36,199	35,671	98.51

### 3.2 Analysis of dilution curve and rank abundance curve

**Figure 1:**
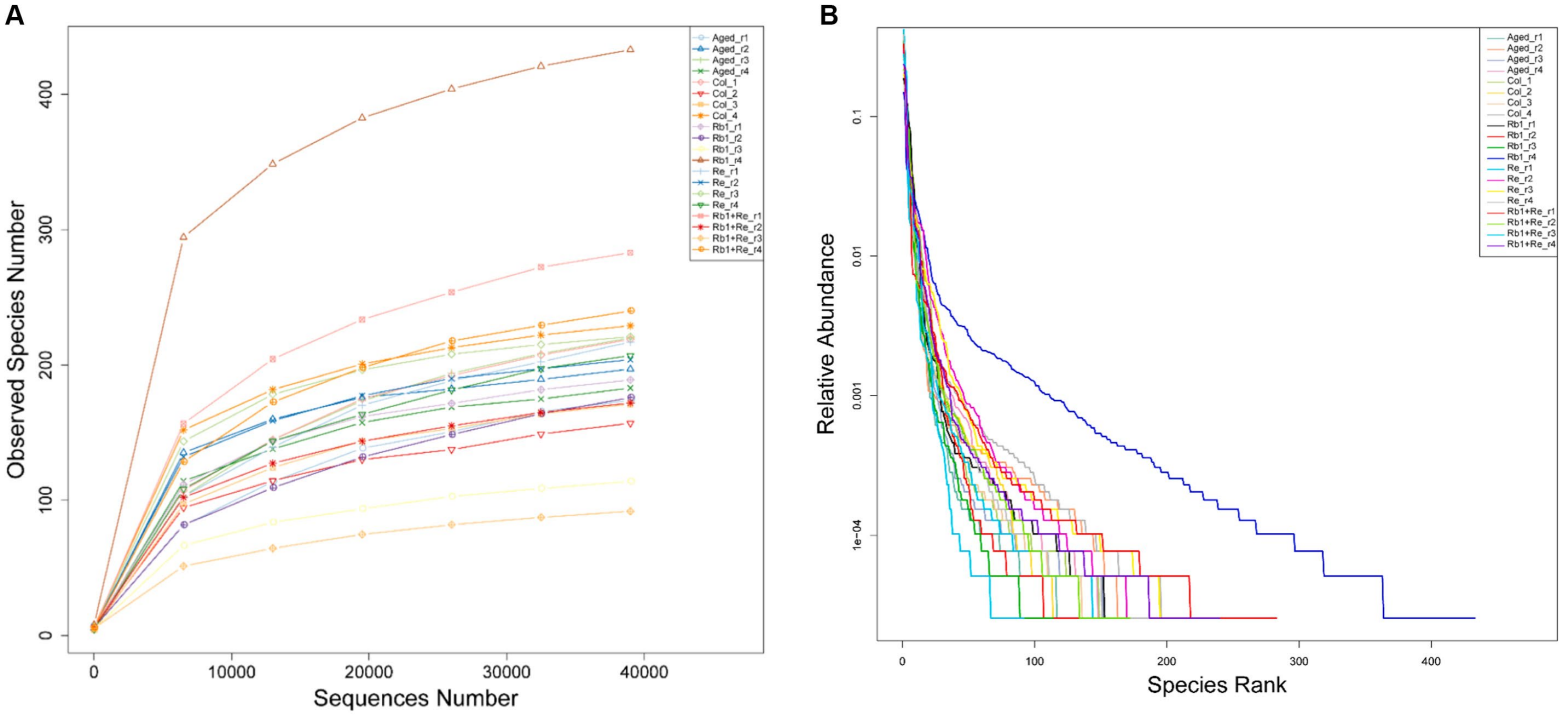
16s rarefaction curves and the rank-abundance distribution. **(A)** Within-sample richness, estimated by OTU analysis of 16S sequences with QIIME. The rarefaction curves are computed by sampling the number of 16S sequences indicated on the x-axis and counting the number of distinct observed species, plotted on the *y*-axis. The shaded areas around the lines represent the 95% confidence intervals. None of the confidence intervals overlap, suggesting that each group significantly differs from the other two even when only 10,000 sequences are considered. **(B)** The horizontal axis in the figure is the serial number of each OTU, and the vertical axis is the relative abundance count of bacteria in this category.

Based on sequencing data, draw dilution curves to compare the species richness of different sequencing samples, and can also be used to demonstrate whether the sequencing data of the samples is sufficient. The dilution curve results show that the sequencing data tends to plateau after exceeding 20,000, reaching a plateau period, indicating that the sequencing data volume for each sample is sufficient (Figure 1A).

The Rank Abundance curve can be used to explain two aspects of diversity, namely species abundance and community evenness. In the horizontal direction, species richness is reflected by the width of the curve, and the higher the species richness, the larger the range of the curve on the horizontal axis; The smoothness of the curve reflects the evenness of species in the sample, and the flatter the curve, the more uniform the distribution of species in the community (Figure 1B) (33, 34).

In summary, the dilution curve and Rank Abundance curve analysis results show that all data are sufficient, and the diversity richness and diversity of OTUs in the sample are moderate, which are consistent with the characteristics of general gut microbiota research. Therefore, this sequencing data can be well used for the analysis of gut microbial diversity.

### 3.3 Alpha diversity analysis

The results of Alpha diversity analysis showed that there was no significant difference in Simpson index among the groups, but Chao1 and Shannon indices showed that the R_b1_, R_e_, and R_b1_ + R_e_ groups had higher total species numbers, and the microbial diversity of the Rb1 + Re group returned to the Col level; In addition, there was no significant difference in ACE values between the Col, R_b1_, R_e_, and R_b1_ + R_e_ groups, but they were all significantly higher than the Aged group. In summary, the administration of R_b1_ and R_e_ alone can significantly increase the abundance of intestinal bacteria, while the administration of R_b1_ and R_e_ has a more significant effect. This indicates that saponins R_b1_ and R_e_ have a significant impact on gut microbiota, and their combined use is crucial for the restoration of gut microbiota diversity (Table 3).

**Table 3:** Alpha diversity analysis.

	Shannon	Simpson	Chao1	Ace
Aged	3.246^c^	0.821	220.972^b^	225.631^b^
Col	3.633^ab^	0.829	232.85^a^	240.011^a^
R_b1_	3.484^b^	0.869	212.129^b^	228.953^ab^
R_e_	3.529^b^	0.819	210.482^b^	239.519^a^
R_b1_ + R_e_	3.856^a^	0.841	243.889^a^	239.819^a^

The same letter indicates no significant statistical difference (*p* >0.05), while different letters indicate significant statistical difference (*p* <0.05).

### 3.4 Beta diversity analysis

**Figure 2:**
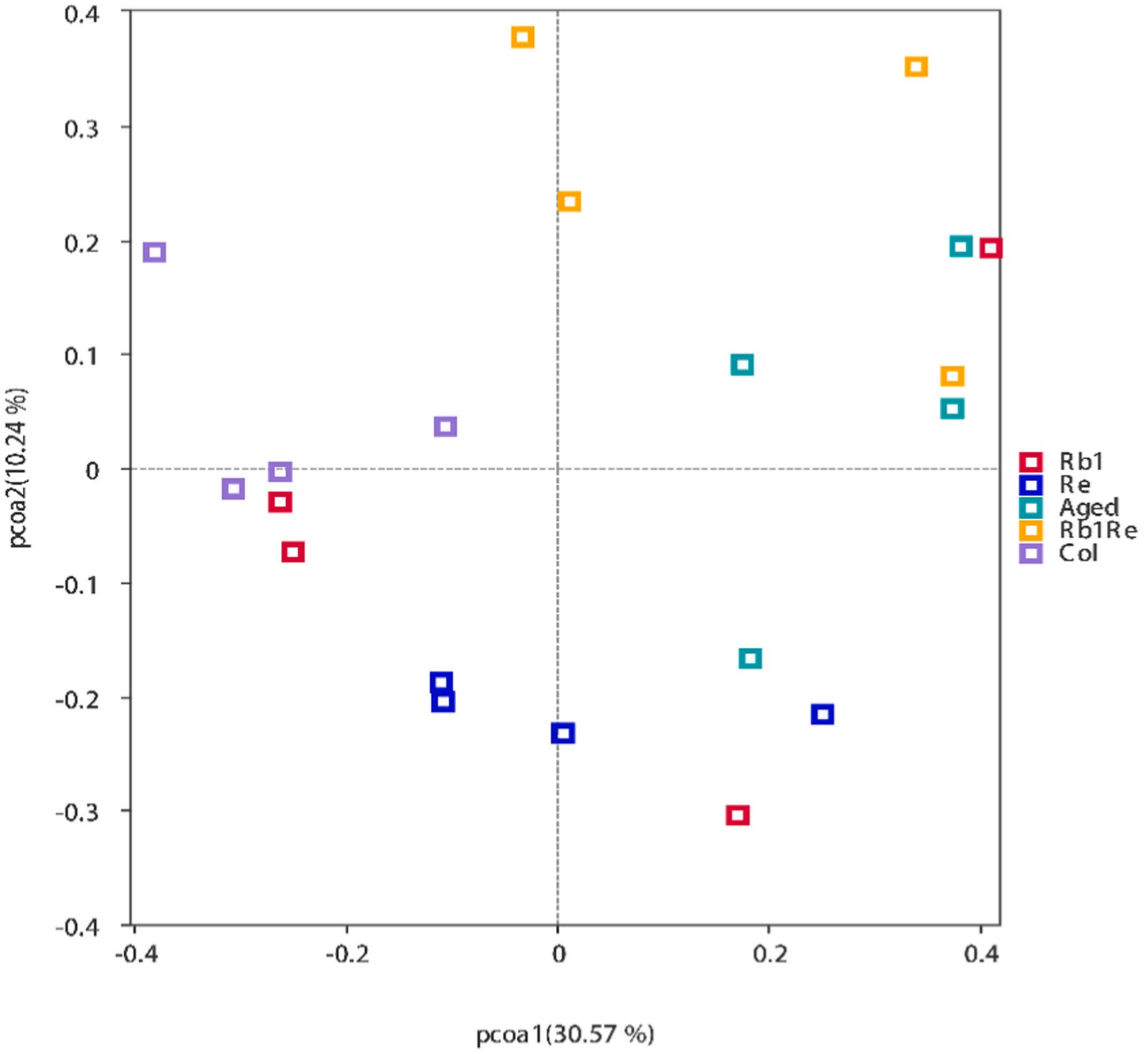
Principal Coordinates Analysis (PCoA) results showing the relatedness of microbial communities in the different samples. The PCoA plots were constructed with the unweighted UniFrac PCoA method.

The Beta diversity analysis results show that the samples within each group can be clustered together. Meanwhile, this study found that the samples from the Col group, Re group, and most of the R_b1_ group were distributed in the second, third, and fourth quadrants. The R_b1_ + R_e_ and most of the Aged group samples are mostly distributed in the first quadrant. This indicates that the Col group, R_e_ group, and R_b1_ group may have similar diversity; the diversity of R_b1_ + R_e_ group and Aged group is relatively similar (Figure 2).

### 3.5 Bar chart of relative species abundance

#### 3.5.1 Differences in gut microbiota abundance at the phylum level

This study analyzed the classification of gut bacteria at the phylum level and the abundance of gut bacteria at the genus level, respectively. Firstly, this study analyzes gut microbiota at the phylum level. The results showed that Firmicutes, Proteobacteria, Teneriicutes, Actinobacteria, Bacteroidetes, Cyanobacteria, Fusobacteria, Acidobacteria, and Candida were among the top 10 bacteria. In the Col, R_b1_, R_e_, and R_b1_ + R_e_ groups, Firmicutes had the highest abundance; In the aging model group, Proteobacteria had the highest abundance of bacteria. In addition, the relative abundance of Tennericutes was very low in the Aged and R_b1_ + R_e_ groups, but higher in the Col, R_b1_, and R_e_ groups.

**Figure 3:**
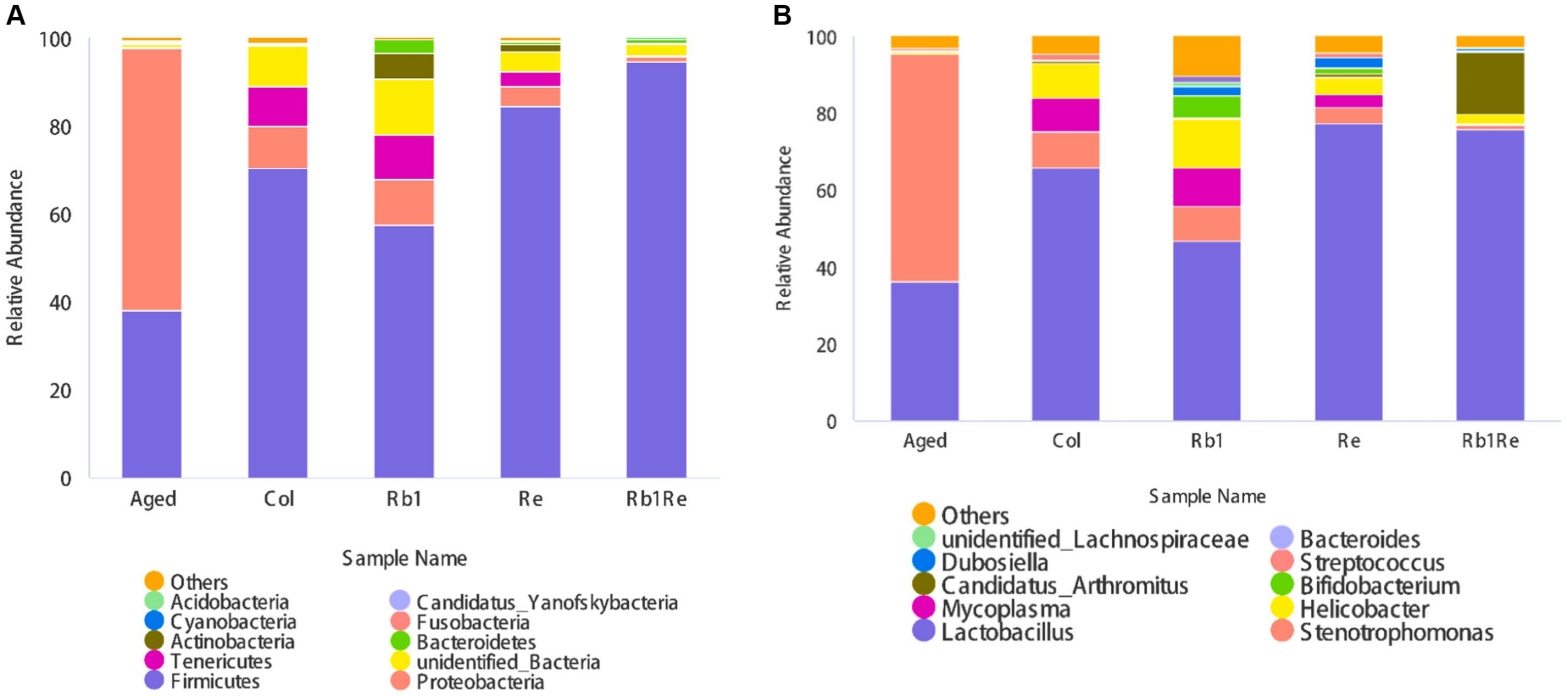
Relative abundance of gut microbiota in different groups. **(A)** Relative abundance of total intestinal microorganisms in different groups at the phylum level. **(B)** Relative abundance of total intestinal microorganisms in different groups at the genus level. The abscissa is grouping, and the ordinate is relative abundance (%).

In addition, we found a significant increase in the relative abundance of Actinobacteria in the R_b1_ and R_e_ intervention groups, but a lower abundance in the R_b1_ + R_e_ group (Figure 3A).

#### 3.5.2 Differences in gut microbiota abundance at the genus level

At the genus level, Lactobacillus, Stenotrophomonas, Helicobacter, Candidatus, Bifidobacterium, Dubosella, Streptococcus, unclassified Lachnospiraceae, and Bacteroides are among the top 10 bacteria in terms of relative abundance.

At the phylum level, Lactobacillus is the most abundant gut bacterium among the Col, R_b1_, R_e_, and R_b1_ + R_e_ groups. However, in the aging model group, the abundance of Lactobacillus was lower than that of Stenotrophomonas, ranking second. The results of this study showed that R_b1_ or R_e_ intervention alone significantly reduced the abundance of Stenotrophomonas (*p* < 0.05). The effect of simultaneous administration is more significant.

In addition, there is a higher proportion of Helicobacter species in the Col group, which may cause gastrointestinal disorders. However, the abundance of Helicobacter in the aging model is actually lower. However, after administration of R_e_ or R_b1_ + R_e_, the abundance of Helicobacter species decreased compared to the Col group, but remained higher than Aged. In addition, this study also found a significant increase in the abundance of *Clostridium difficile* (Candidatus Arthromitus) in the R_b1_ + R_e_ group, while the abundance of *Clostridium difficile* (Candidatus Arthromitus) was lower in other groups. Candidatus Arthromitus is a collective term for a large group of Gram positive, anaerobic, or microaerophilic crude Bacillus bacteria, mainly found in soil, human and animal intestines, and spoilage, but most are not pathogenic. The co administration of R_b1_ + R_e_ promotes a rapid increase in the abundance of *Clostridium difficile*, indicating that the co intervention of R_b1_ + R_e_ may provide a more suitable living environment for *Clostridium difficile*. This change may be directly caused by changes in organic acids in the intestine. The co administration of R_b1_ and R_e_, with a larger dosage and longer course of treatment, may lead to changes in organic acids in the intestine. Lactobacillus and Bifidobacterium are recognized probiotics.

The results of this study showed that after treatment with R_b1_ or R_e_, the abundance of Lactobacillus significantly increased, reflecting the effect of saponin monomers on intestinal probiotics. In addition, the intervention of R_b1_ significantly increased the abundance of Bifidobacterium, while the intervention of R_e_ had a certain promoting effect on Bifidobacterium, but it was far less effective than that of R_b1_. This suggests that R_b1_ may provide a more suitable environment for gut bifidobacteria in aging mice. The simultaneous use of R_b1_ and R_e_ has limited impact on the abundance of Bifidobacterium (Figure 3B).

### 3.6 LEfSe analysis

**Figure 4:**
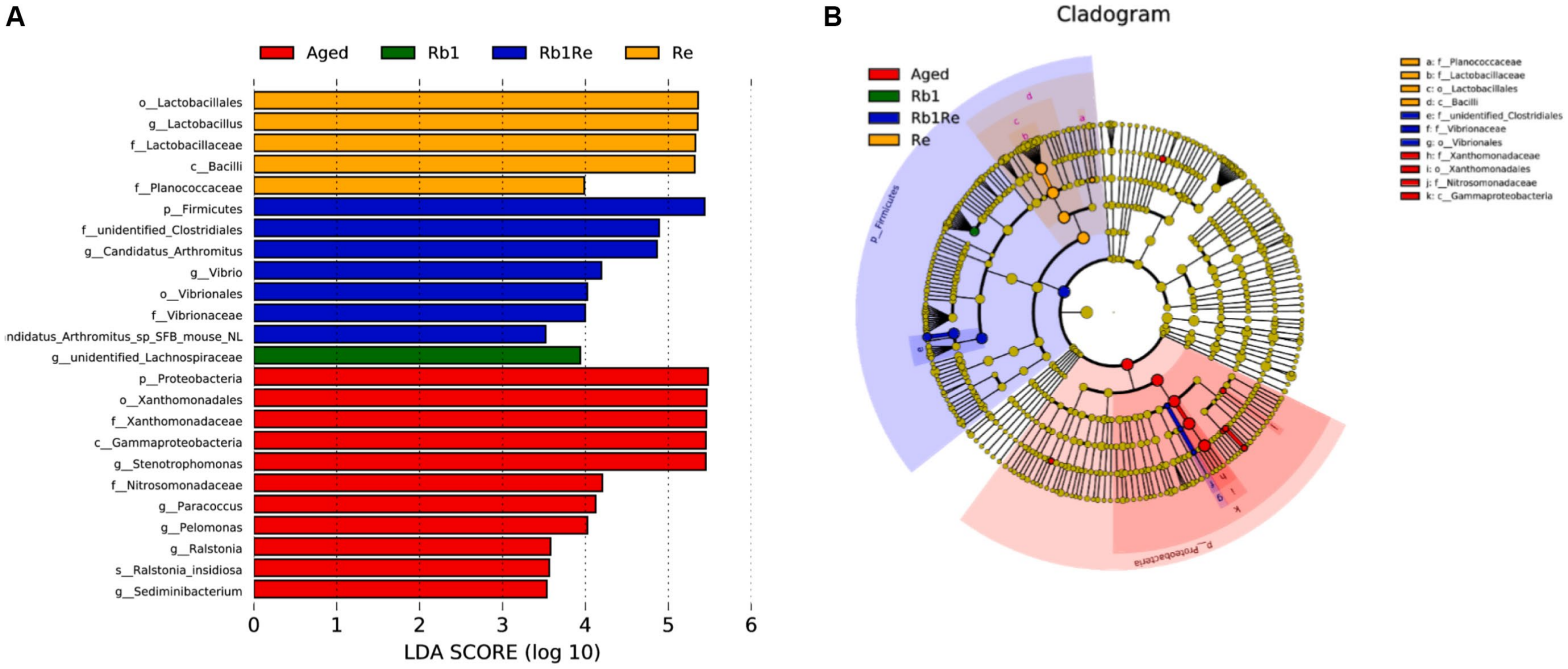
LEfSe analysis. **(A)** Bacterial biomarkers found by linear discriminant analysis effect size (LEfSe). **(B)** Taxonomic cladogram derived from LEfSe analysis of 16S sequences. Taxa meeting a linear discriminant analysis significant threshold >3.5 are shown. c, class level; f, family level; g, genus level; o, order level; p, phylum level; LDA, Linear discriminant analysis.

This study used LEfSe analysis to screen for bacterial species with significant differences in abundance between groups. The results showed that in the Aged group, there was a significant difference in abundance among the most diverse bacterial species, while R_b1_ had the least diversity among the other groups (Figures 4A,B). Meanwhile, this study found no significant differences in bacterial species between the Col group and the other groups. Lactobacillales, Lactobacillaceae, and Lactobacillus genera have a significant impact in the R_e_ group, indicating that R_e_ may be one of the main factors causing changes in the abundance of lactobacilli in the gut microbiota. Simultaneously using R_b1_ and R_e_ to intervene in an aging mouse model resulted in a significant increase in gut Firmicutes. In addition, the abundance of Vibrio in the R_b1_ + R_e_ group significantly increased compared to other groups. Vibrio genus contains a large number of pathogenic bacteria, such as classical and Elto biotypes of *Vibrio cholerae* and *Vibrio parahaemolyticus*. Higher levels of Vibrio may cause diarrhea. This indicates that the combined use of R_b1_ and R_e_ may have certain negative effects on the gut of aging individuals. In this study, Proteobacteria in the Aged group had the highest abundance among all groups. As is well known, Proteobacteria is the largest phylum of bacteria, including many pathogenic bacteria such as *Escherichia coli*, Salmonella, *Vibrio cholerae*, *Helicobacter pylori*, and other famous species. After administration of R_b1_ or R_e_, the abundance of Proteobacteria significantly decreased, indicating that R_b1_ or R_e_ can significantly inhibit the abundance of Proteobacteria related pathogenic bacteria in the intestine. However, the simultaneous use of the two may have new issues.

### 3.7 OTU functional clustering analysis

**Figure 5:**
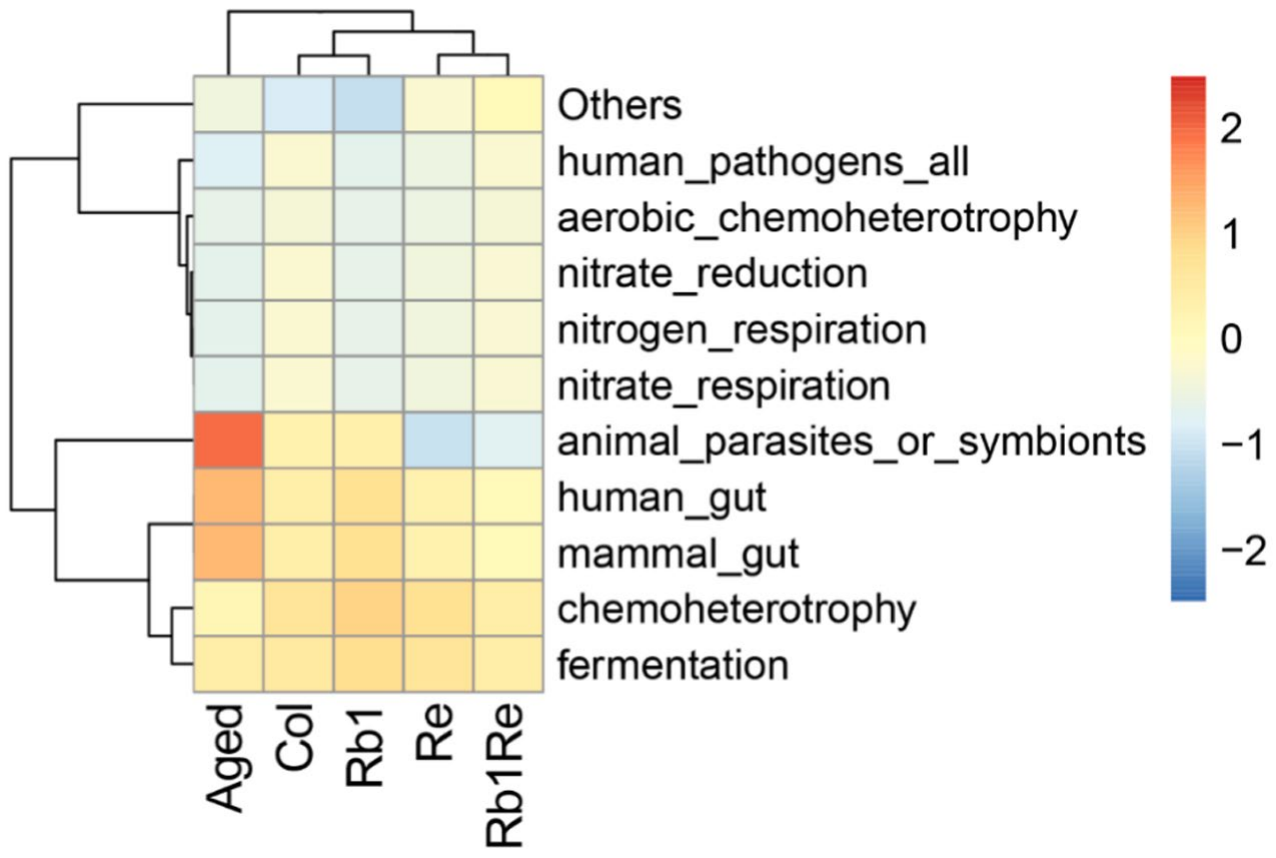
OTU function cluster. Predicting microbial community functions. The redder the color indicates higher abundance of the microbial community under that function, while the bluer the color indicates lower abundance of the microbial community under that function.

According to the clustering results, it was found that the Aged model group had a higher abundance of animal parasites or symbionts. Based on the results, the study identified genes involved in the parasite’s survival and adaptation to its host, as well as their potential to cause disease. In the case of symbiotes, functional enrichment analysis can reveal the unique metabolic pathways and processes of these organisms, and contribute to the symbiotic relationship with the host. In the aging model, there may be a higher risk of diseases caused by parasitic organisms, while in the Col group, this risk is significantly reduced. Furthermore, after intervention with R_b1_ and R_e_, this risk is further reduced. Especially with R_e_ intervention, the risk of diseases caused by parasitic organisms was significantly reduced, and there was a significant difference compared to the aging group and the control group (Figure 5). In addition, this study found that OTUs related to human gut and mammal gut have more similar abundances in the Col, R_b1_, R_e_, and R_b1_ + R_e_ groups, forming a significant difference from the Aged group. Therefore, it can be inferred that after R_e_ and R_b1_ intervention, the gut microbiota pattern is closer to that of the Col group, greatly improving the gut microbiota status of the aging model.

### 3.8 Microbial-environmental factor redundancy analysis

Aging negatively affects both motor and cognitive performance. In recent years, the study of gut microbiome and the gut-brain axis and its neural functions has received extensive attention, so the homeostasis of gut microbiome is closely related to the recovery of cognitive function in aging mice. Studies have found that the imbalance of intestinal microecology is associated with Alzheimer’s Disease (AD), Parkinson’s Disease (PD), Huntington’s Disease (Huntington’s disease), and Alzheimer’s disease. HD is closely related to neurological disorders such as Multiple Sclerosis (MS). The team previously assessed the mice’s ability to adapt to new environments through open field and water maze experiments. Among them, the water maze experiment studied the learning and memory ability of mice. Saponins have obvious restorative effect on the degraded motor ability and spatial cognitive ability of aging individuals. The exercise ability of aging mice decreased significantly compared with control group. The supplement of Re may be helpful to restore part of the motor ability of aging mice to a certain extent. Re or Rb1 + Re intervention can reduce the rest time of aging mice and increase the slow movement time, which reflects the effectiveness of Re. However, in the case of rapid exercise, neither Re, Rb1 nor Re + Rb1 interventions showed a significant effect. Aging mice had spatial learning and memory impairment, and the escape latency of mice after Rb1 and Re intervention was significantly reduced compared with that of the aging model group, which indicated that Rb1 or Re intervention alone could effectively improve the cognition of the aging model. In addition, simultaneous intervention with Rb1 and Re had better effects. The exploration time of the aging mice platform quadrant in Rb1 or Re treatment group was increased compared with that in aging group, but it was significantly lower than that in control group. The simultaneous intervention of Rb1 and Re did not produce a significant difference from the intervention alone, suggesting that Rb1 and Re do not have a superimposed effect on neurological recovery. Rb1 and Re intervention can effectively improve the number of synapses in the hippocampus, and Rb1 had a better effect than Re. However, simultaneous intervention with Rb1 + Re did not show a greater effect. Rb1 or Re intervention could significantly improve the morphology of microglia, which were mostly round, but the number of lysosomes did not change significantly. Therefore, Rb1 and Re intervention alone or Rb1 + Re intervention together can improve the hippocampus microglia, but cannot alleviate the influence of lysosomes, and the combined intervention of Rb1 and Re cannot form a superimposed effect.

**Figure 6:**
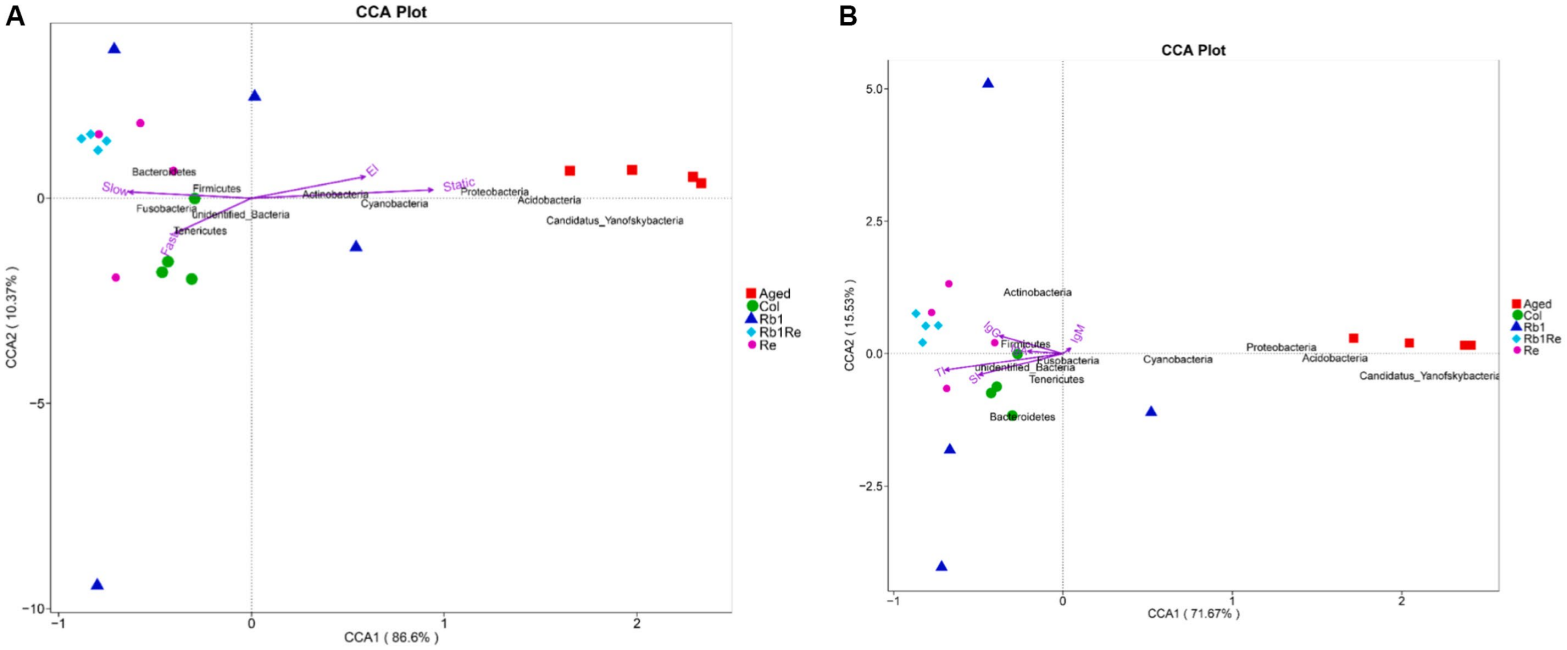
Multivariate redundancy analysis (RDA) of the microbiota composition. **(A)** Exercise and cognition. **(B)** Immune indexes. RDA was performed using Canoco 5.0. Taxonomic composition at the genus level was used as response data, iron level as explanatory variable, time point and experiment as supplementary variables. The variation explained by the ordination axis is significantly higher than random (permutation test).

Through redundancy analysis, the results showed that Aged group was positively correlated with inactivity duration, while Col group was negatively correlated with inactivity duration, indicating that Aged group mice had limited exercise ability and their physical function was far lower than Col group. However, the exercise ability of aging models was significantly improved after R_b1_ or R_e_ administration, which was positively correlated with the slow exercise duration in the R_e_ and R_b1_ + R_e_ groups, indicating that the administration of R_b1_ or R_e_ could improve the exercise ability of aging mice, but could not achieve the same exercise level as Col. The platform quadrant residence time was negatively correlated with Col and each drug treatment group (Figure 6A).

In addition, this study found that the abundance of Proteobacteria, Acidobacteria, Cyanobacteria and Candidatus Yanofskybacteria was positively correlated with quiescence time and platform quadrant residence time. Tenericutes were positively correlated with rapid motor ability. The abundance of Bacteroidetes and Firmicutes is related to slow motion. Although there is no direct evidence that these bacteria are involved in the regulation of motor and cognitive ability in aging models, it can at least be suggested that they are associated with exercise and cognition in aging models. These changes may be caused by Rb1 and Re by regulating the gut-brain axis.

By analyzing the relationship between intestinal microorganisms and immune indexes, we found that the Aged group was rich in Cyanobacteria, Proteobacteria, Acidobacteria and Candidatus Yanofskybacteria. Its abundance was inversely proportional to thymus index and spleen index (Figure 6B). In addition, this study found that Firmicutes were in direct proportion to IgG, and Rb1 + Re group had a higher abundance of Firmicutes. Therefore, this study suggests that Firmicutes may be related to immune indexes in aging models.

### 3.9 Microbial interaction network analysis

**Figure 7:**
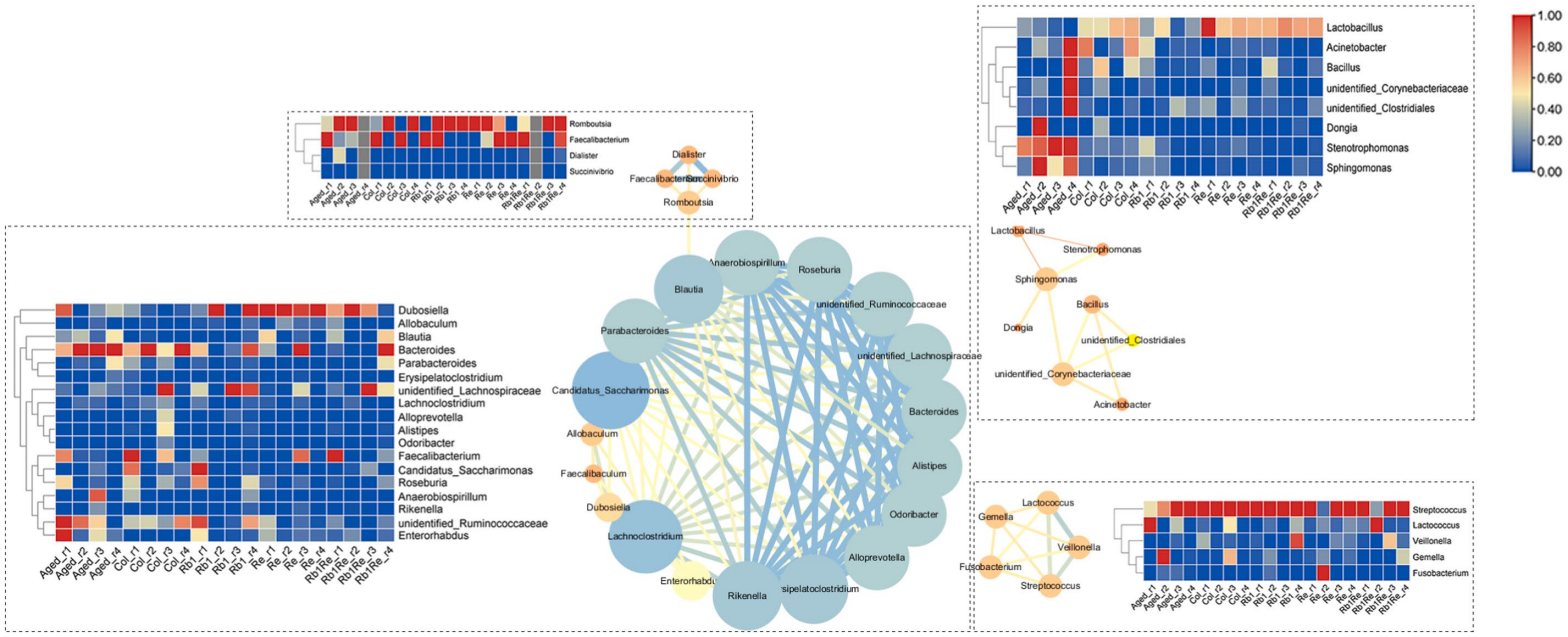
Analysis of microbial community interaction network. The redder the color is, the higher the abundance of the corresponding microbiota is, and the bluer the color is, the lower the abundance of the corresponding microbiota is.

The interaction network analysis of the major intestinal microbes showed that the interactions of these microbiota were divided into four subgroups. Among them, the largest subgroup contains 18 genera, and the interaction is strong. According to the gut microbial abundance analysis, the abundance of Dubosiella increased significantly after R_b1_ and R_e_ intervention, while the abundance of unidentified_Ruminococcaceae decreased significantly after R_b1_ and R_e_ intervention. However, the abundance of these 18 genera was lower than 1%, which was a non-dominant bacterial group. The dominant bacterial group forms the second largest interaction network subgroup, in which Lactobacillus has a high abundance in Col and a low abundance in Aged, but the abundance of Lactobacillus also increases to the same level of Col after R_e_ intervention. In addition, the interaction between Lactobacillus and Stenotrophomonas and Sphingomonas is strong, which may be that the increase of Lactobacillus competes with Stenotrophomonas and Sphingomonas. Stenotrophomonas and Sphingomonas were inhibited in abundance (Figure 7).

## 4 Discussion

Rezaeiasl et al. showed that supplementation with Lactobacillus can have a positive impact on the learning ability of the rat model of Alzheimer’s disease (35). Lactobacillus also improves the gut microbiota in *Drosophila melanogaster* Alzheimer’s disease models (36). So the increase in the abundance of Lactobacillus from R_b1_ and R_e_ reflects its positive effect on the aging model. In addition, R_b1_ and R_e_ suppressed the number of enterobacteriaceae bacteria, particularly Proteobacteria, and increased the number of Lactobacillus and Bifidobacterium. In addition, previous studies have shown that Lactobacillus can inhibit the expression of TNF-α and IL-1β in macrophages, thereby alleviates colitis (37). *Bifidobacterium longum* CH57 alleviates colitis by inhibiting the NF-κB signaling pathway and TNF-α expression (38). *Lactobacillus plantarum* C29 improves colitis in older mice by inhibiting NF-κB signaling (39). Other studies have shown that *Lactobacillus casei* DN-114001 inhibits DSS induced colitis by inhibiting intestinal membrane permeability and NF-κB activation (40).Some probiotics have also been shown to restore gut microbiota composition and fecal lipopolysaccharide levels in mice with colitis. These findings suggest that increased probiotics can reduce colitis symptoms by inhibiting NF-κB activation and restoring disrupted gut microbiota composition. In addition, these probiotics significantly inhibited blood lipopolysaccharide and TNF-α levels in mice with TNBS induced colitis. Therefore, the intervention of R_b1_ and R_e_ in this study led to an increase in the number of intestinal probiotics, and the impact on the aging model may be multifaceted, requiring more experimental verification.

The aging process is accompanied by a gradual decline in body structure and function. Older adults often experience a number of health-related problems, such as insomnia, anxiety, depression, and cognitive decline. These problems may be related to changes in gut microbes and disturbances in the brain-gut axis. The brain-gut axis refers to the interaction between the digestive tract and the central nervous system. Gut microbes can influence neurotransmission and function in the brain by secreting metabolites such as short-chain fatty acids and neurotransmitters. The nervous system influences the growth and metabolism of intestinal microbes by regulating the movement and secretion of the gut. This two-way interaction is important for the health of the human body.

According to the results of this study, there may be a high risk of diseases caused by parasitic organisms in the aging model, and the risk is further reduced in the Col group and R_b1_ + R_e_ intervention group, indicating that the main components of saponins can significantly improve the intestinal microbiota status of the aging model. In addition, this study found that the abundance of Proteobacteria, Acidobacteria, Cyanobacteria and Candidatus Yanofskybacteria was positively correlated with quiescence time and platform quadrant residence time. Tenericutes were positively correlated with rapid motor ability. The abundance of Bacteroidetes and Firmicutes is related to slow motion. These changes may be achieved through the brain-gut axis, but this requires more work to prove one by one.

Gut microbiota is a complex microecosystem in the host body, and its stability is closely related to body health (41). The gut microbiome composition and the communication between the microbiome and the brain may change with age, and the microbiome composition of older people over 65 years of age is significantly different from that of a 9-year-old child (42–44). Intestinal symptoms, loss of motor ability, low immunity, and even the occurrence of neurological diseases such as Parkinson’s disease and Alzheimer’s disease have been observed in aging populations (45–47). Previous studies have confirmed that nervous system diseases are closely related to the composition of intestinal microbiota, which may be related to the fact that bacterial metabolites, such as short-chain fatty acids, can mediate the maturation of microglia (48), and may also affect the clearance of Aβ to affect the effect on neurons (49). Studies have shown that when aging rats are infected with *E. coli*, its Aβ deposition in intestinal and brain neurons increases, and the proliferation of glial and astrocytes is enhanced (50).

When antibiotics were used to interfere with the gut microbial diversity of Alzheimer’s mice, they showed significant reductions in Aβ deposition and glial cell proliferation (51). Therefore, there is a clear correlation between the gut microbes of mice and their cognitive ability. In this study, R_b1_ and R_e_ changed the intestinal microbiome profile of aging mouse models, and R_b1_ and R_e_ reduced Proteobacteria, indicating that the abundance of many pathogens, such as *Escherichia coli*, Salmonella, *Vibrio cholerae* and *Helicobacter pylori*, were inhibited by R_b1_ or R_e_. And this has a definite positive effect on the recovery of the nervous system of the aging model. Therefore, R_b1_ and R_e_ may influence the nervous system of aging models through the brain-gut axis through the regulation of gut microbes.

In addition, changes in the gut microbiota are not dominated by one or a few dominant microbiota. The interactions of individual microbiota are equally important in the gut microbiome. As we age, the number and diversity of gut microbes may change, causing the balance and interaction of microbes to break down. This imbalance can lead to intestinal inflammation and decreased immune function. At the same time, the increased permeability of the intestinal wall in the elderly may cause bacteria and toxins to enter the blood circulation, triggering an inflammatory response. In this study, a complex interaction regulatory network was formed among all kinds of bacteria. Other studies have shown that diet and probiotic intake can improve the balance of intestinal microbes and help prevent diseases such as cognitive decline and depression in the elderly (52). Combined with the results of this study, the supply of R_b1_ and R_e_ may have a positive effect on the abundance of probiotics.

In summary, the relationship between aging, the brain-gut axis, and gut microbes is very strong. By maintaining the balance of intestinal microbes and the normal function of brain-gut axis, saponins can improve the intestinal microbiota of aging individuals, and make the body obtain intestinal microbial homeostasis closer to that of young healthy mice.

## 5 Conclusion

R_b1_, R_e_, and R_b1_ + R_e_ all significantly increased intestinal microbiota diversity, and R_b1_ + R_e_ showed a better effect than R_b1_ or R_e_ alone. The simultaneous intervention of R_b1_ and R_e_ can restore the intestinal microbiota diversity to the level of young mice. The increase in the abundance of probiotics, Lactobacillus and Bifidobacterium after R_b1_ and R_e_ intervention reflects its positive effect on aging mice, and its inhibitory effect on the number of enterobacteriaceae, such as Proteobacteria bacteria. Through the prediction of OTU function, the results showed that the risk of parasitic diseases in aging mice was further reduced when R_b1_ and R_e_ were simultaneously interfered, and there was a significant difference between the aging group and the control group. The abundance of Proteobacteria, Acidobacteria, Cyanobacteri and Candidatus Yanofskybacteria was positively correlated with stationary time and platform quadrant residence time. The abundance of Tenericutes was positively correlated with the ability to move rapidly. The abundance of Bacteroidetes and Firmicutes is related to slow motion. In addition, the abundance of Cyanobacteria, Proteobacteria, Acidobacteria and Candidatus Yanofskybacteria was negatively correlated with thymus index and spleen index. The abundance of Firmicutes was positively correlated with IgG. Although there is no direct evidence to prove that these microflora are directly involved in the regulation of motor ability, cognitive ability and immunity in aging mice, it can at least show that there is a correlation between gut microflora and movement, cognition and immunity in aging models.

## Data Availability

The raw data supporting the conclusions of this article will be made available by the authors, without undue reservation.
